# Influence of advanced maternal age (≥35 years) on maternal and neonatal outcomes in vaginally intended breech deliveries at term—A FRABAT study

**DOI:** 10.1002/ijgo.70689

**Published:** 2025-11-24

**Authors:** Bilal Alemi, Tatjana Nobs, Samira Catharina Hoock, Eileen Deuster, Anna Elisabeth Hentrich, Anne Kristina Kämpf, Wiebke Schaarschmidt, Frank Louwen, Lukas Jennewein

**Affiliations:** ^1^ “Frankfurt Breech at Term (FRABAT) Collective”, Department of Obstetrics and Perinatal Medicine University Hospital Frankfurt, Goethe University Frankfurt Frankfurt Germany

**Keywords:** advanced maternal age, breech, FRABAT, vaginal breech delivery

## Abstract

**Objective:**

To examine maternal and neonatal outcomes in women aged 35 years and older compared with those under 35 years following planned vaginal breech deliveries.

**Methods:**

This study encompassed all vaginally intended breech deliveries at the Department of Obstetrics and Perinatal Medicine at Goethe University Hospital in Frankfurt from January 2004 to December 2023 (Frankfurt Breech at Term Cohort). Neonatal and maternal outcomes were compared by dividing the women into two groups and analyzing differences between groups using multivariate nominal regression adjusted for parity.

**Results:**

No difference in perinatal morbidity (measured with a modified PREMODA score) was observed for women with advanced age (35 years and older) (odds ratio [OR] 1.50, 95% confidence interval [CI] 0.74–3.02, *P* = 0.257) in vaginally intended deliveries. We found a significant association with advanced maternal age and cesarean section (OR 1.50, 95% CI 1.21–1.86, *P* < 0.001). Analysis of vaginally completed deliveries showed higher odds of postpartum hemorrhage (OR 1.50, 95% CI 1.19–5.25, *P* = 0.015).

[Correction added on 20 December 2025 after first online publication: The preceding paragraph was corrected.]

**Conclusion:**

Maternal age of 35 years or older is not linked to higher perinatal morbidity following vaginally attempted breech delivery. Nonetheless, a detailed consultation before delivery remains essential, particularly when pre‐existing health conditions are present.

**Deutsches Register klinischer Studien:**

DRKS00025030 (https://drks.de/search/de/trial/DRKS00025030/details)

## 1 INTRODUCTION

The optimum management of breech presentation at term remains a critical issue in obstetric care throughout the world, with significant implications for maternal and neonatal outcomes if not managed properly by experienced personnel in specialized centers. Although external cephalic version and elective cesarean delivery have become standard approaches, vaginal breech deliveries have again found recognition in national guidelines.^1^, ^2^, ^3^, ^4^

Hence, understanding the factors influencing maternal and fetal outcome in breech presentation at term is essential for informed clinical decision making and risk assessment.

One factor, known for its impact on pregnancy and peripartum outcome, is advanced maternal age (AMA), commonly defined as pregnancy occurring in women aged 35 years or older. Over the past decades, the prevalence of pregnancies in women of AMA has increased globally, driven by sociocultural and economic trends that support delayed childbearing.^5^ AMA is associated with various obstetric challenges, including an increased risk of chromosomal anomalies,^6^ stillbirth,^7^, ^8^ cesarean delivery,^9^ and placenta previa.^10^ However, its potential role in influencing perinatal outcomes in breech presentation at term has been less explored in depth.

Breech presentation, characterized by the fetus's buttocks or feet being positioned to deliver first, occurs in approximately 3%–4% of term pregnancies. Abnormal placentation, including placenta accreta spectrum disorders, may predispose to malpresentation.^11^, ^12^ After publication of the Term Breech Trial^13^ cesarean section became the common approach when managing breech presentation. Due to its controversial nature, multiple other studies were published, highlighting the topic and leading to heightened clinical interest in identifying modifiable and non‐modifiable risk factors. The influence of AMA on breech presentation is hypothesized to be multifactorial, encompassing changes in uterine compliance, parity, and associated obstetric conditions. Despite this, the relationship between AMA and breech presentation at term has not been consistently documented, leaving a critical gap in evidence that may influence prenatal counseling and clinical interventions.

This research aims to investigate the outcomes among term pregnancies planning vaginal breech birth, with a focus on elucidating potential underlying mechanisms and influences and evaluating the clinical implications of these findings. By leveraging robust data and contemporary statistical methods, the present study seeks to contribute to the growing body of literature addressing the complexities of AMA and its impact on pregnancy outcomes. The findings may inform tailored obstetric care strategies, particularly in populations experiencing an upward trend in maternal age.

## 2 MATERIALS AND METHODS

### 2.1 Study design

A retrospective analysis based on a prospectively maintained registry cohort of all term singleton breech presentations at the Department of Obstetrics and Perinatal Medicine, Goethe University Hospital, Frankfurt, was conducted between January 2004 and December 2023.

The study cohort included two groups of which the first comprised pregnant women below the age of 35 years with a young maternal age (YMA) while the second comprised those from the age of 35 years (i.e. AMA).

All patients with breech presentation at term who selected a vaginal delivery approach and did not fulfill any exclusion criteria were included.

Exclusion criteria for vaginal delivery were defined as major uterine abnormalities, extensive fibroid removals, intertuberous distance less than 11 cm, an estimated fetal birth weight below 2500 g, or fetal malformations complicating vaginal delivery. Planned cesarean sections due to the patient's wish were also excluded.

Neonatal morbidity was assessed using a modified version of the PREMODA (prematurity and morbidity in breech delivery at term) composite score. This score was chosen because it is a validated instrument specifically designed for evaluating outcomes in term breech deliveries. The original PREMODA criteria encompass neonatal death, intubation lasting more than 24 h, neonatal intensive care unit (NICU) admission exceeding 4 days, a 5‐min Apgar score less than 4, seizures occurring after the first 24 h, and birth trauma (brachial plexus injuries, parietal skull fractures, humerus and femur fractures, and cervical soft‐tissue injuries). For the present analysis, the PREMODA score was adapted to exclude morbidities unrelated to the mode of delivery (e.g. congenital malformations, infections) and to include cases of documented neurologic deficits. These criteria capture severe and clinically relevant neonatal morbidity and have been employed in previous FRABAT (Frankfurt Breech at Term collective) studies,^14^, ^15^, ^16^ allowing direct comparability with the original PREMODA cohort reported by Goffinet et al.^17^ while enhancing clinical relevance.

The primary outcome was neonatal morbidity in successful vaginal breech deliveries, defined according to the modified PREMODA composite score. Secondary outcomes included cesarean section rate and maternal complications.

The university hospital's ethics committee consented to the study protocol (Reference number 2021‐126). The assessment of obstetric medical records was carried out after the discharge of patients using the hospital patient management system, an electronic database (GeDoWin®, Saatmann GmbH) and a state database called the *Perinatalerhebung Hessen*. Data were anonymized. All patients were treated within standard clinical care. Patients consented in writing because the study was set up as a registry study.

### 2.2 Clinical approach

Between 34 and 36 weeks of pregnancy every patient with a breech presentation is counseled regarding vaginal delivery and cesarean section.^14^, ^18^ An external cephalic version is always offered unless contraindications (e.g. uterine malformations, amniotic band) exist. The procedure is usually performed in week 38 of pregnancy and the probability of success is discussed with the patient.^19^

After thorough elaboration of the delivery mode, a shared decision is reached.

In primipara, magnetic resonaonce imaging (MRI) to determine the obstetric conjugate and intertuberous distance is conducted. Patients with an intertuberous distance of less than 11 cm are advised against a vaginal approach whereas those with an obstetric conjugate less than 12 cm are informed that the risk of an emergency cesarean section is elevated.^20^

As maternal and fetal outcome is favorable in an upright birth position, this is generally recommended.^18^ An upright position implies that the patient either stands or is positioned on the knees with the delivery bed's upper part being elevated. The birth is accompanied by a senior physician and potential maneuvers to help delivering the fetal arms or head are carried out by the attending physician.^16^ Labor induction was conducted in accordance with national guidelines and followed the same procedures as those applied for cephalic presentation.

### 2.3 Statistical analysis

With continuous variables, normal distribution was tested using Kolmogorov–Smirnov testing. For normal distributed variables, *t*‐testing was used to analyze differences. The analysis of group differences with non‐parametric values was performed using Pearson *χ* ^2^ test and Fisher exact test. Variables with potential confounding activity (parity, epidural, comorbidities) were analyzed using multivariate nominal logistic regression in several approaches to test for confounding. Because parity is an important influencing factor in obstetrics and delivery outcome data, all following examinations were performed using nominal logistic regression adjusting for parity (Tables 2, 3, 4). Odds ratio (OR) with 95% confidence interval (CI) and *P* value (Walds testing) were calculated. Statistical analyses were carried out with JMP software (SAS Institute, Cary, NC, USA) and a *P* value of less than 0.05 was deemed statistically significant.

## 3 RESULTS

The FRABAT cohort of women at the Goethe University Hospital, Frankfurt, during the period of January 2004 to December 2023 with a breech presentation at term (≥37 weeks of pregnancy) included 3097 pregnancies. 2029 attempted vaginal delivery and 998 women underwent an elective cesarean section. Seventy cases were excluded because of incomplete data.

**FIGURE 1:**
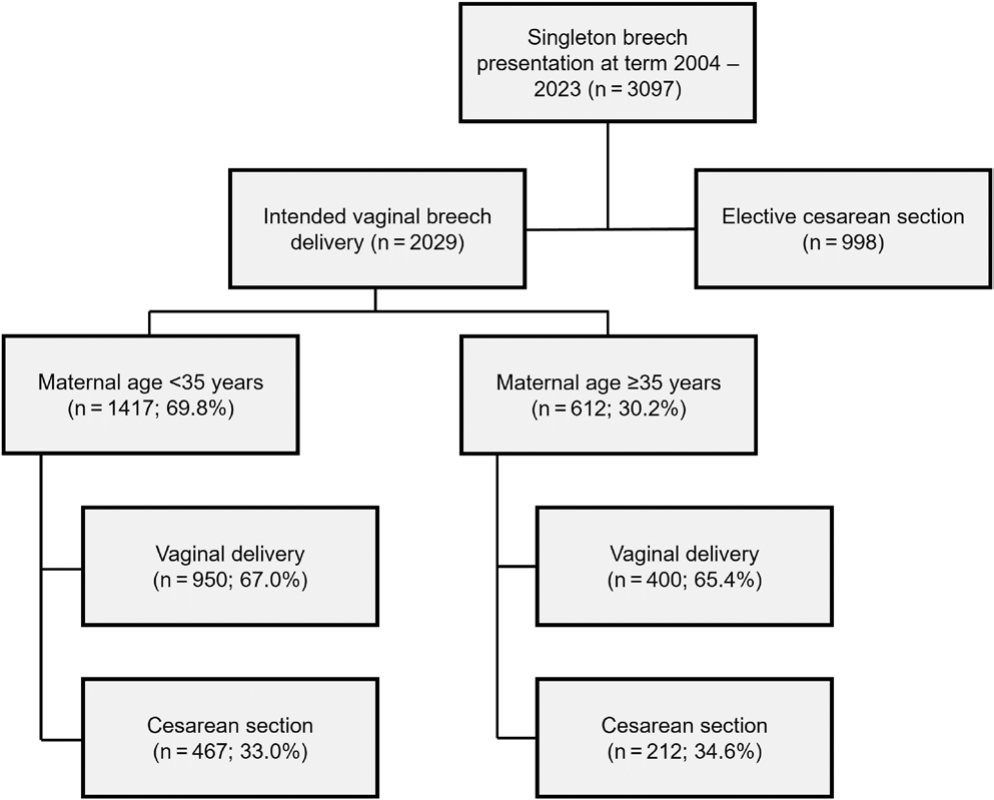
Flowchart of study group.

This analysis comprises the 2029 pregnancies involving vaginally planned term breech presentations, stratified by maternal age into less than 35 years (YMA group; *n* = 1417; 69.8%) and 35 years or older (AMA group; *n* = 612; 30.2%). Of the 2029 planned vaginal approaches, 1350 women eventually delivered vaginally (Figure 1). The mean age of the YMA group was 30.3 ± 3.1 years while the AMA group averaged 37.2 ± 2.3 years. The body mass index (calculated as weight in kilograms divided by the square of height in meters) as well as the duration of pregnancy were equally distributed in both groups (Table 1). Advanced maternal age was associated with significantly higher proportion of multiparous women in the AMA group (41.3% versus 33.6%, *P* < 0.001). Additionally, women in the AMA group were more likely to have pre‐existing conditions (22.6% versus 16.7%, *P* = 0.002). Pre‐existing conditions were defined as conditions known or diagnosed before pregnancy and relevant to pregnancy and/or delivery (Table 1). Furthermore, a significantly higher percentage of minor abnormal uterine conditions (7.5% versus 3.7%, *P* < 0.001) could be noted in the AMA group. Minor uterine conditions comprised previous cesarean section, small fibroids, previous myomectomy without extensive uterus reconstruction or uterine cavity opening, and minor uterine abnormalities (arcuate uterus, small uterine septum).

**TABLE 1:** Vaginally intended deliveries out of a breech position at term, epidemiologics and maternal patient history, for women with maternal age < 35 versus age ≥ 35 years.^a^

Characteristic	Age < 35 years (*n* = 1417)	Age ≥ 35 years (*n* = 612)	*P* value
Age, y	30.3 ± 3.1	37.2 ± 2.3	<0.001
Body mass index^b^	23.0 ± 3.9	23,4 ± 4.0	0.034
Duration of pregnancy, wk	39.9 ± 1.2	39.9 ± 1.2	0.83
Parity			<0.001
Primipara	941 (66.4%)	359 (58.7%)	
Multipara	476 (33.6%)	253 (41.3%)	
Gestational diabetes	77 (5.4%)	46 (7.5%)	0.2
Pre‐existing conditions^c^	236 (16.7%)	138 (22.6%)	0.002
Hemostasis disorder	33 (2.3%)	22 (3.6%)	0.107
Type of breech			
Frank	651 (68.5%)	258 (64.5%)	0.15
Complete	126 (13.3%)	41 (10.3%)	0.125
Incomplete	104 (11%)	59 (14.8%)	0.05
Minor uterine abnormalities^d^	53 (3.7%)	56 (7.5%)	<0.001

^a^ Data are presented as mean ± standard deviation or as number (percentage).

^b^ Missing body mass index values (calculated as weight in kilograms divided by the square of height in meters): 36 patients.

^c^ Including pre‐existing hypertension, diabetes, anemia, obesity.

^d^ Small fibroids, previous myomectomy without extensive uterus reconstruction or uterine cavity opening, minor uterine abnormalities (arcuate uterus, small uterine septum).

Group differences in maternal and neonatal outcomes were further assessed using nominal logistic regression models adjusted for parity (Tables 2, 3, 4).

**TABLE 2:** Vaginally intended deliveries out of breech presentation at term, fetal outcome, for women with maternal age < 35 versus age ≥ 35 years.

Nominal logistic regression independent variable: age ≥ 35 y, adjusted to parity	OR	95% CI	*P* value (Walds testing)
Cesarean section	1.50	1.21–1.86	<0.001
Epidural analgesia	0.89	0.73–1.09	0.257
Apgar score at 5 min <4	2.05	0.63–6.67	0.235
NICU >4 days	0.90	0.56–1.43	0.649
Intubation	1.35	0.50–3.65	0.554
pH arterial blood <7.0	1.00	0.25–4.06	0.994
Birth trauma; excluding hematoma^a^	1.28	0.41–3.98	0.665
Neurologic deficits	0.58	0.12–2.78	0.497
Perinatal morbidity (PREMODA)	0.92	0.59–1.43	0.727
Perinatal morbidity potentially related to birth mode^b^	1.50	0.74–3.02	0.257

Abbreviations: CI, confidence interval; NICU, neonatal intensive care unit; OR, odds ratio.

^a^ Registered birth traumas were transient plexus paralysis and fractures of the clavicle, humerus, or femur.

^b^ Perinatal morbidity included cases of asphyxia (18), severe adjustment disorder (12), plexus paralysis after assisted delivery of arms (6), humerus fracture after assisted delivery of arms (2), clavicular fracture (2), femur fracture (1). There was no perinatal death. All newborns were discharged in good clinical condition.

Advanced maternal age was associated with cesarean section (OR 1.50, 95% CI 1.21–1.86, *P* < 0.001) (Table 2). There was no significant association of AMA and epidural analgesia (OR 0.89, 95% CI 0.73–1.09, *P* = 0.257, Table 2). To measure fetal outcome, we used the above described modified PREMODA score, which was previously published by our group.^17^ Nominal logistic regression demonstrated that AMA was not associated with increased perinatal morbidity potentially related to birth mode (OR 1.50, 95% CI 0.74–3.02, *P* = 0.257) in vaginally intended deliveries. Likewise, no significant associations were found for Apgar less than 4 at 5 min, NICU longer than 4 days, intubation, arterial pH less than 7.0, birth trauma, or neurologic deficits (Table 2). Among completed vaginal breech deliveries, regression analyses again revealed no significant age‐related differences in perinatal outcomes potentially linked to birth mode (OR 1.73, 95% CI 0.79–3.77, *P* = 0.171).

Following adjustment for parity, AMA was not significantly associated with epidural analgesia use in vaginal birth (OR 0.84, 95% CI 0.66–1.09, *P* = 0.189), but it showed higher odds of postpartum hemorrhage (OR 1.50, 95% CI 1.19–5.25, *P* = 0.015). Perineal injury (OR 1.08, 95% CI 0.85–1.38, *P* = 0.526), high‐grade perineal tear (OR 0.55, 95% CI 0.18–1.67, *P* = 0.294), and episiotomy (OR 1.46, 95% CI 0.67–3.16, *P* = 0.340) did not differ significantly between age groups (Table 4).

Among vaginal deliveries (*n* = 1350), the type of breech presentation did not differ between age groups (Table 5). Complete and incomplete breech presentations are reported for consistency with previous FRABAT analyses.^14^, ^16^

## 4 DISCUSSION

In recent years, many studies have approached the topic of maternal age and potentially resulting adverse outcomes in pregnancy and childbirth.^21^, ^22^, ^23^, ^24^ However, research concerning the relationship of AMA, breech presentation, and perinatal outcome is limited. Our findings provide valuable insights into the relationship between maternal age and the safety of planned vaginal breech delivery, a subject of critical importance in modern obstetric care given the rising trend of pregnancies among older women^5^ and the linear correlation between breech presentation and maternal age.^25^

To account for potential confounding, especially that due to differences in parity distribution between groups, all outcome analyses were performed using nominal logistic regression adjusted for parity in our study. This allowed for a more robust assessment of independent associations between AMA and obstetric outcomes.

The primary observation from our study is that AMA is not associated with an increase in perinatal morbidity or mortality in vaginally planned breech deliveries at term. Using the PREMODA criteria^17^ (Tables 2 and 3), we found no significant differences between the YMA group (<35 years) and the AMA group in terms of short‐term neonatal morbidity.

**TABLE 3:** Vaginal deliveries out of breech presentation at term, fetal outcome, in women with maternal age < 35 versus age ≥ 35 years.

Nominal logistic regression independent variable: age ≥ 35 y, adjusted to parity	OR	95% CI	*P* value (Walds testing)
Manual assistance	0.94	0.74–1.21	0.653
Apgar score at 5 min <4	1.91	0.53–6.93	0.323
NICU >4 d	0.75	0.39–1.45	0.398
Intubation	1.84	0.60–5.65	0.285
pH arterial blood <7.0	3.23	0.61–17.31	0.169
Birth trauma; excluding hematoma	1.49	0.47–4.72	0.496
Neurologic deficits	0.97	0.19–4.94	0.971
Perinatal morbidity (PREMODA)	0.82	0.45–1.49	0.512
Perinatal morbidity potentially related to birth mode	1.73	0.79–3.77	0.171

Abbreviations: CI, confidence interval; NICU, neonatal intensive care unit; OR, odds ratio.

**TABLE 4:** Vaginal deliveries out of breech presentation at term, maternal outcome, in women with maternal age < 35 versus age ≥ 35 years.

Nominal logistic regression independent variable: age ≥ 35 y adjusted to parity	OR	95% CI	*P* value (Walds testing)
Perineal injury	1.08	0.85–1.38	0.526
High‐grade perineal tear (III°/IV°)	0.55	0.18–1.67	0.294
Episiotomy	1.46	0.67–3.16	0.340
Epidural analgesia	0.844	0.66–1.09	0.189
Postpartum hemorrhage^a^	1.5	1.19–5.25	0.015

Abbreviations: CI, confidence interval; OR, odds ratio.

^a^ Postpartum hemorrhage of ≥500 mL blood loss after vaginal delivery or ≥1000 mL blood loss after cesarean section.

**TABLE 5:** Vaginal deliveries out of breech position at term, type of breech, in women with maternal age < 35 versus age ≥ 35 years.^a^

Characteristic	<35 years (*n* = 950)	>35 years (*n* = 400)	*P* value
Type of breech			
Frank	651 (68.5%)	258 (64.5%)	0.15
Complete	126 (13.3%)	41 (10.3%)	0.125
Incomplete	104 (11%)	59 (14.8%)	0.05

^a^ Data are presented as number (percentage).

Interestingly, AMA was associated with higher odds of cesarean section (OR 1.50, 95% CI 1.21–1.86), even after adjustment for parity. This observation is consistent with evidence demonstrating an age‐related rise in cesarean deliveries and identifying maternal age as an independent determinant of operative birth.^9^ The higher cesarean likelihood among older mothers in our cohort may therefore reflect both physiologic factors influencing labor progression and a more cautious intrapartum approach adopted by clinicians when managing AMA patients. Nevertheless, despite the increased surgical rate, neonatal morbidity remained unaffected, underscoring that planned vaginal breech delivery can be safely pursued in appropriately selected women under experienced supervision.

It is important to highlight that neonatal morbidity in breech deliveries largely depends on the adherence to best practices and the skill level of the attending team. For both younger and older mothers, meticulous planning, including the assessment of fetal weight, type of breech presentation, and maternal pelvic dimensions, likely mitigated risks. Especially the management with an upright maternal position during delivery and enabling women to be moving in an upright position, e.g. with walking epidural,^15^ can ensure a safe vaginal breech delivery.^18^ These findings underscore the need for individualized prenatal counseling and close monitoring during delivery, particularly for breech presentation.^15^

In adjusted analyses, epidural analgesia use did not differ significantly between age groups (OR 0.84, 95% CI 0.66–1.09), suggesting that the apparent difference observed in unadjusted comparisons was largely driven by parity distribution. Conversely, our results show significantly higher odds of postpartum hemorrhage in AMA women (OR 1.50, 95% CI 1.19–5.25), suggesting that AMA may independently increase the risk of uterine atony or impaired contractility. This finding contrasts with some previous studies that did not identify maternal age as an independent predictor of postpartum hemorrhage.^9^, ^26^, ^27^ The discrepancy may reflect differences in cohort characteristics or obstetric management strategies, such as the reserved use of oxytocin being part of the clinical protocol. Physiologically, age‐related alterations in myometrial function and vascular remodeling could contribute to a reduced contractile response during the third stage of labor, warranting heightened vigilance and proactive management in AMA patients. Perineal injuries, including first‐ and second‐degree tears, were similar across age groups, and severe tears (third‐ and fourth‐degree) remained rare, with no statistically significant differences. This differs from previous observations regarding AMA and cephalic presentation.^28^ The low rate of episiotomies in both groups reflects a conservative approach to their use, aligning with modern obstetric guidelines that advocate (super‐)selective, rather than routine, episiotomy for vaginal deliveries.^29^

Another noteworthy finding is the higher proportion of incomplete breech presentations in the AMA group. This may reflect underlying differences in uterine compliance or fetal positioning influenced by maternal age. Despite this, incomplete breech presentations did not significantly impact maternal or neonatal outcomes, indicating that such cases can be managed effectively with proper care.

The AMA group exhibited a higher prevalence of pre‐existing conditions, in line with the established correlation between advancing maternal age and increased comorbidities. Moreover, AMA is associated with a higher incidence of gestational hypertension, diabetes mellitus, and pre‐eclampsia. Given that obesity, an increasing global health concern, is frequently associated with these conditions, its role as a contributing factor warrants consideration.^27^

Although the mean body mass index values were comparable across the study cohorts, the global rise in obesity may pose additional risks for pregnant women in the future.^30^ These conditions pose additional challenges in breech deliveries and underscore the importance of thorough pre‐delivery assessments in older women. This study reinforces the importance of comprehensive prenatal counseling for women of AMA considering vaginal breech delivery. Counseling should address the potential risks and benefits of both vaginal and cesarean deliveries, considering anatomical and fetal factors,^31^, ^32^ maternal age and pre‐existing conditions. For women with breech presentations, shared decision making and individualized care plans are crucial to optimizing outcomes.

Over the 20‐year study period, management of term breech delivery evolved substantially. Following publication of the Term Breech Trial^13^ toward planned cesarean section, there was a marked global decline in vaginal breech births and a temporary loss of intrapartum expertise.^33^ Subsequent evidence demonstrating the safety of vaginal breech birth in selected women and renewed training initiatives^18^ supported a structured re‐introduction of the procedure. During this evolution, the upright or all‐fours birthing position progressively replaced the traditional lithotomy posture, improving maternal biomechanics and neonatal outcomes.^18^ In parallel, our institution transitioned from X‐ray to MRI pelvimetry for assessing pelvic adequacy.^20^ Although MRI pelvimetry provides accurate, radiation‐free assessment of pelvic dimensions, its limited availability internationally must be recognized as a limitation when comparing our results to studies relying on clinical or radiographic assessment alone. Advancements in analgesia practice and neonatal care, including refined NICU admission criteria, likely contributed to evolving outcome definitions. Collectively, these secular changes emphasize the importance of accounting for calendar period in future FRABAT analyses and interpreting the present findings within an evolving clinical and infrastructural framework. Our findings also highlight the need for specialized training in vaginal breech delivery techniques, particularly as the reintroduction of this option gains traction in national guidelines.^1^, ^2^, ^3^, ^4^ Clinicians should be well‐versed in managing complications, such as incomplete breech presentations and postpartum hemorrhage, which are more common in our cohort of AMA women. Our data demonstrate that, even after statistical adjustment for parity, AMA alone is not an independent contraindication for planned vaginal breech delivery at term. However, the higher adjusted odds of cesarean section and postpartum hemorrhage highlight that specific intrapartum risks persist and require tailored management. These insights emphasize the importance of both individualized clinical assessment and the continued availability of experienced obstetric teams in centers offering vaginal breech birth. The use of 35 years as the cut‐off for AMA may lead to inconsistencies when comparing our findings with studies that apply a higher age threshold. Historically, 35 years has been identified as the cut‐off for AMA because of the noted increase in chromosomal abnormalities starting at this age.^6^, ^34^ However, it is crucial to view this association as a continuum, with age‐related risks increasing progressively rather than abruptly.^10^, ^35^ Consequently, delivery complications reported in studies with a higher age cut‐off should be interpreted with caution, as they may be more closely related to maternal age itself rather than the fetal presentation.

Contrary to some studies reporting higher cesarean rates in older mothers, predominantly regarding cephalic presentation,^9^, ^35^, ^36^ our data revealed no significant differences in cesarean section rates between the YMA and AMA groups. This discrepancy may be attributed to our study's focus on planned vaginal deliveries where careful patient selection likely minimized the need for emergency interventions.

The study benefits from a large sample size and a robust data set spanning nearly two decades. The inclusion of a single‐center cohort ensures consistency in clinical protocols, and the stratification of outcomes by maternal age provides meaningful insights into the influence of AMA on breech deliveries.

A major strength of this study is the use of multivariate nominal logistic regression to adjust for key confounders, thereby enhancing the validity of the associations observed. However, given the retrospective design, other unmeasured variables such as provider experience, patient preference, and subtle selection biases may still have influenced the observed relationships and limits the ability to draw causal conclusions.

Additionally, as this study was conducted in a tertiary care setting, the findings may not be generalizable to hospitals with lower levels of expertise in managing breech deliveries. The exclusion of patients who opted for planned cesarean delivery also introduces potential selection bias, as women with higher risk profiles may have been advised to choose cesarean delivery pre‐emptively.

Future research should explore the long‐term outcomes of neonates delivered vaginally in breech presentations, with a focus on potential age‐related differences in developmental trajectories. Also, prospective studies incorporating diverse healthcare settings would enhance the generalizability of findings and provide a broader understanding of the role of maternal age in breech deliveries.

Further studies investigating the molecular and biomechanical changes associated with aging uterine tissue could highlight the mechanisms underlying the observed differences in outcomes between younger and older mothers. Such research could contribute to the development of targeted interventions to optimize care for women of AMA.

In conclusion, AMA does not inherently predispose women to higher perinatal morbidity in planned vaginal breech deliveries when appropriate patient selection and standardized management protocols are applied. Nevertheless, AMA is associated with distinct maternal challenges, including a greater burden of pre‐existing medical conditions, an elevated risk of postpartum hemorrhage, and higher odds for cesarean section. These factors underscore the need for individualized counseling and vigilant intrapartum management by experienced obstetric teams. As the trend toward delayed childbearing continues, these findings hold particular relevance for optimizing clinical decision making and informing evidence‐based policies regarding the management of breech presentations in women of AMA.

## AUTHOR CONTRIBUTIONS

BA contributed to manuscript writing, editing and data collection. TN contributed to manuscript review, editing and data collection. AKK, WS, AEH, SCK, ED contributed to manuscript review and editing. FL was responsible for protocol/project development, manuscript review and editing. LJ performed protocol/project development, data analysis, and manuscript writing and editing.

## FUNDING INFORMATION

No funding was received.

## CONFLICT OF INTEREST STATEMENT

The authors have no conflicts of interest.

## Data Availability

Research data are not shared.
